# Multiple bilateral breast hematomas as a complication of polyacrylamide hydrogel injection for breast augmentation: A case report

**DOI:** 10.1016/j.jpra.2025.08.039

**Published:** 2025-09-05

**Authors:** Houhuang Qiu, Qian Liang, Xiang Zhou, Fuqiang Pan

**Affiliations:** a Department of Medical Cosmetology, Second Affiliated Hospital of Guangxi Medical University, No. 166 Daxue East Road, Xixiangtang District, Nanning City, Guangxi Province, China; b Shenzhen Nanshan People's Hospital, No. 89 Taoyuan Road, Nanshan District, Shenzhen City, Guangdong Province, China

**Keywords:** Breast hematomas, Polyacrylamide hydrogel, Injection for breast augmentation

## Abstract

We present the case of a female patient who underwent polyacrylamide hydrogel (PAAG) injection for breast augmentation 21 years ago and developed bilateral breast pain 2 years prior to evaluation. Preoperative magnetic resonance imaging (MRI) did not reveal any hematoma formation. However, intraoperative findings included multiple mural masses composed of dark red, fibrous clots, which were subsequently diagnosed as chronic organized hematomas. This case illustrates that organized hematoma may represent a distinct subtype of PAAG-related complications, likely resulting from long-term interactions between the physicochemical properties of PAAG and the local tissue microenvironment. It also highlights the diagnostic limitations of imaging in complex PAAG-associated pathology, suggesting that imaging should serve as an adjunct rather than a sole determinant in surgical decision-making. For patients with persistent pain or morphological changes of the breast, surgical exploration should be considered even in the absence of characteristic imaging findings. For asymptomatic patients, surgical intervention may also represent a beneficial option.

## Introduction

Since its introduction in the 1990s, polyacrylamide hydrogel (PAAG) has been extensively applied in cosmetic surgery practices across Ukraine as a non-biological soft tissue filler, owing to its perceived biocompatibility and the technical simplicity of injection procedures.^1^ PAAG received approval from the China State Food and Drug Administration in 1997 as a permanent injectable filler for aesthetic purposes. Over time, with its growing clinical use, an increasing number of postoperative complications were recognized and documented. Reported adverse outcomes included infections, implant displacement, localized nodules or indurations, breast asymmetry, and diffuse tissue firmness, all of which have posed substantial long-term risks to patient health. Within the decade following its market entry, it is estimated that approximately 200,000 Chinese women underwent breast augmentation using PAAG injections. Due to mounting concerns over its safety profile, Chinese health authorities officially prohibited its use in cosmetic surgery in 2006.^2^^,^^3^

The management of complications arising from PAAG injections poses significant clinical challenges, largely due to the material’s unpredictable behavior, pronounced tissue diffusibility, and its propensity for extensive migration. Currently, comprehensive surgical debridement is regarded as the cornerstone of treatment. Surgical strategies must be individualized based on specific clinical indications and the extent of tissue involvement, and may include PAAG extraction, capsulectomy, targeted lesion excision, or mastectomy when warranted.^4^

## Case report

The patient, a female, underwent bilateral breast augmentation with PAAG injections at an outside facility 21 years prior; the volume of injected material was not recorded. Approximately 2 years ago, she began reporting episodic bilateral breast discomfort, described as paroxysmal distending pain that worsened during nighttime. These symptoms were accompanied by noticeable alterations in breast contour. She denied any nipple discharge, cutaneous erythema, ulceration, or systemic symptoms such as fever.

In the seated position, the patient’s right breast measured approximately 14.0 × 14.0 × 10.0 cm, while the left measured about 16.5 × 14.0 × 16.0 cm. Chest circumference was recorded at 99.0 cm. Notable asymmetry was observed, with the left breast appearing larger than the right. Both breasts were firm on palpation and exhibited no mobility. The nipples were misaligned, with the left positioned higher relative to the right. The overlying skin showed no evidence of erythema, ulceration, or inflammatory changes. Nipple inversion and discharge were absent. No enlarged lymph nodes were palpable in the axillary or supraclavicular regions.

### Investigations

**Figure 1:**
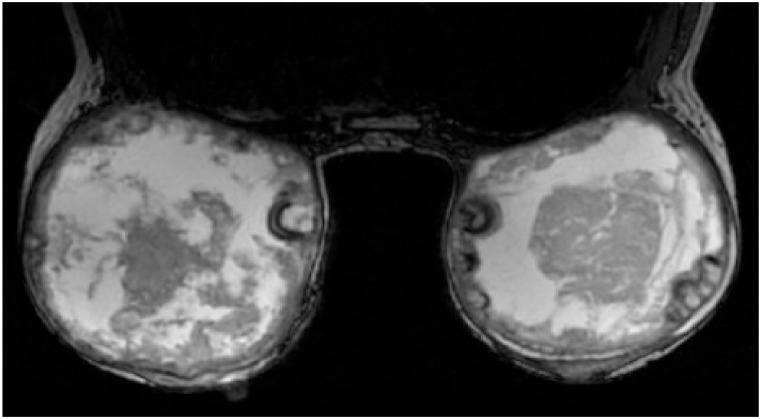
MRI of the patient’s breasts. On In-Phase axial T2 FSE-IDEAL ASSET imaging, the lesion demonstrated a mixed signal pattern with slightly hyperintense and hypointense components, with indistinct margins. Contrast-enhanced scanning revealed marked enhancement, and the time-signal intensity curve exhibited a plateau pattern.

Inflammatory parameters, including white blood cell count, C-reactive protein (CRP), and procalcitonin (PCT), were found to be within normal reference ranges. Tumor markers such as carcinoembryonic antigen (CEA) and carbohydrate antigen 15-3 (CA15-3) showed no evidence of abnormality. MRI demonstrated postoperative alterations related to bilateral breast implant placement. The implants were situated within the retromammary space, posterior to the mammary gland and anterior to the pectoralis major muscle, displaying heterogeneous signal characteristics. Multiple nodular formations with associated calcifications were identified around the implants, raising suspicion for inflammatory granulomas (Figure 1).

### Surgical treatment

**Figure 2:**
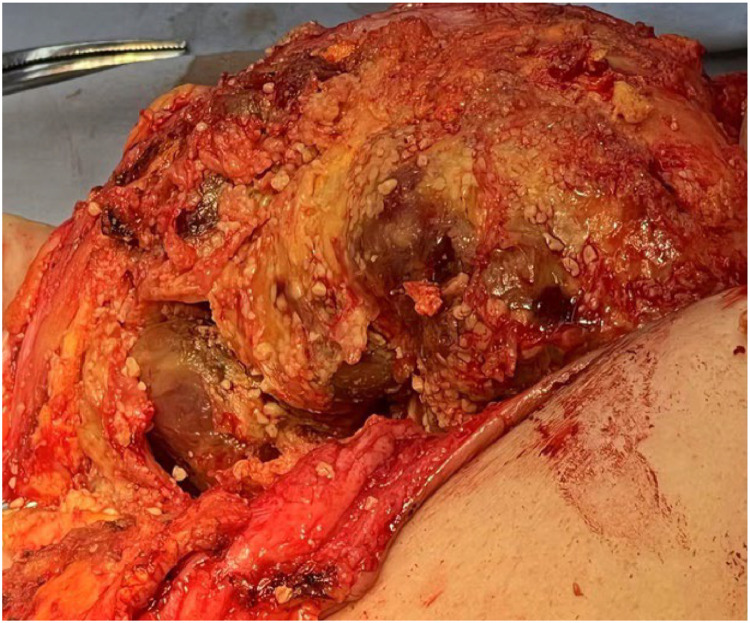
Surgical images. Numerous firm and immobile nodules and masses extensively adhered to the inner capsule wall.

Bilateral semicircular incisions were initially performed along the lower margins of the areolae. Upon breaching the capsule, a substantial volume of dark yellow fluid was released, accompanied by numerous yellowish-white granular deposits, some forming conglomerated masses. Following thorough evacuation of these foreign materials, the injection-induced cavities were carefully explored. Palpation revealed numerous firm, immobile nodules and masses extensively adherent to the inner capsule wall. To ensure complete capsule excision, additional radial incisions approximately 6 cm in length were made in the upper outer quadrants of both breasts (Figure 2). The capsules were meticulously dissected and fully mobilized, with meticulous hemostasis achieved. One drainage tube was placed in each breast. Postoperatively, the breast contours were restored, and the surgical sites were closed with compressive dressings to conclude the procedure.

**Figure 3:**
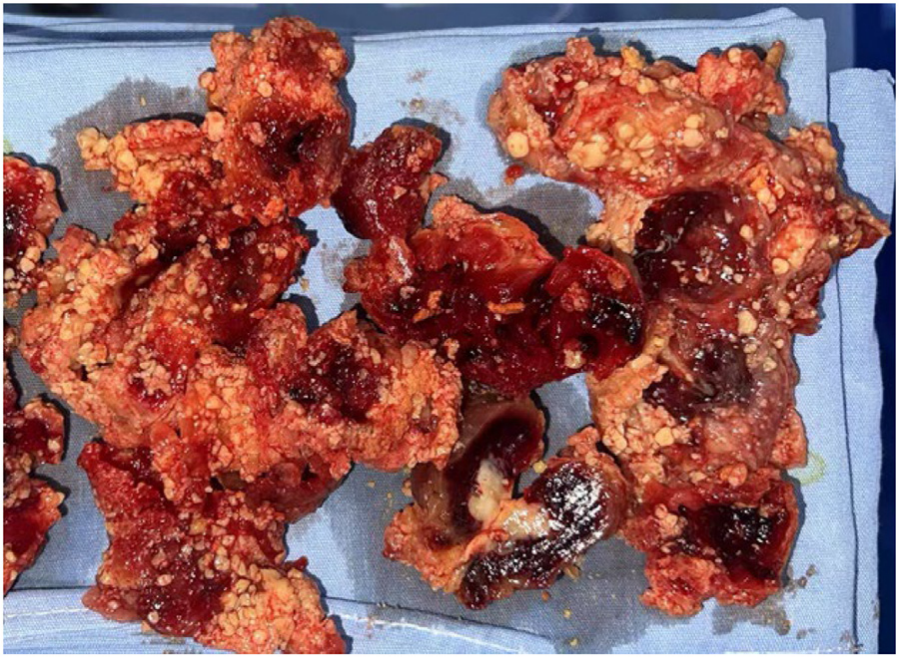
Macroscopic appearance of the capsule and associated mass. Resected mass predominantly consisting of dark red, firm blood clots with a central yellow-white region.

**Figure 4:**
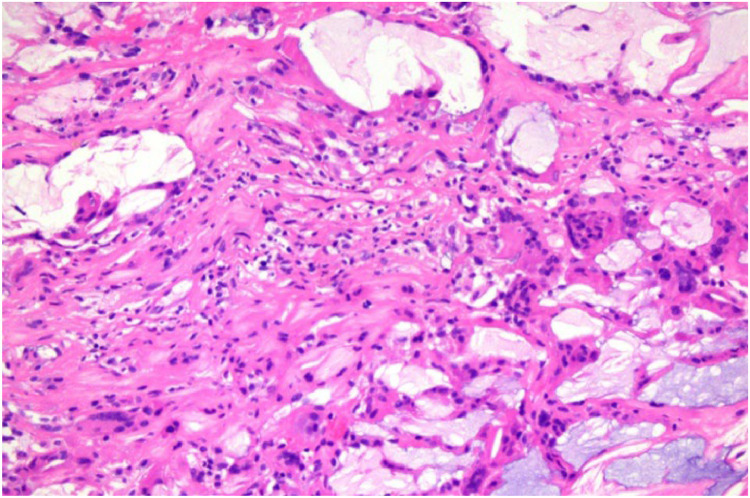
Histopathological findings. Microscopic analysis demonstrates extensive amorphous basophilic deposits with calcifications, surrounded by proliferative fibrous tissue, alongside variable inflammatory cell infiltration and multinucleated giant cell responses.

Gross examination of the excised capsular masses revealed cut surfaces composed predominantly of dark red, firm blood clots with central yellowish-white areas (Figure 3), indicative of hematoma organization and fibrosis. Based on intraoperative observations and specimen characteristics, these masses were diagnosed as long-standing organized hematomas. Histopathological analysis of the capsule demonstrated abundant amorphous basophilic deposits with calcifications. Surrounding fibrous tissue proliferation was evident, accompanied by variable inflammatory cell infiltration and multinucleated giant cell reactions (Figure 4).

## Discussion

Previous studies have indicated that degradation products of PAAG may initiate a sustained chronic inflammatory cascade, primarily driven by continuous macrophage infiltration and cytokine release. This prolonged immune activation can result in progressive tissue fibrosis, peripheral nerve injury, and maintenance of a persistently oxidative microenvironment at the injection site. The oxidative stress, in turn, enhances the production of proteolytic enzymes and reactive oxygen species, exerting corrosive and cytotoxic effects that may ultimately contribute to structural and functional damage of adjacent tissues and organs.^5^^,^^6^ The insidious progression of PAAG-induced chronic inflammation may explain the extended asymptomatic period observed in affected individuals. In the present case, the intraoperative identification of multiple mural hematomas adherent to the fibrous capsule is presumed to result either from inflammatory degradation of vascular structures or from mechanical disruption caused by the frictional movement of migrated PAAG material. Unlike traumatic hematomas, these lesions appear to arise from the complex interplay between the physicochemical properties of PAAG and the host tissue microenvironment. Hematoma may constitute a distinct subtype of complications following PAAG injection for breast augmentation. To date, reports addressing this specific complication remain scarce, and further clinical data are required to support its characterization.

Pain and alterations in breast morphology often serve as the initial clinical manifestations and are closely linked to increased capsular tension and nerve compression resulting from PAAG migration. The absence of elevated inflammatory markers highlights the non-infectious etiology of such complications, which are frequently misinterpreted as neoplastic processes. In the present case, MRI revealed multiple calcified nodular lesions surrounding the implant; however, it failed to distinguish between organized hematoma and granulomatous changes. For patients with complex PAAG-related complications, imaging should be regarded as a supportive diagnostic modality rather than the definitive criterion for surgical planning. When symptoms such as pain or breast deformity arise, surgical exploration should be strongly considered, even in the absence of definitive imaging findings.^3^ The radial incision provided adequate exposure of the fibrous capsule, enabling precise capsular dissection and restoration of the native breast contour, thereby avoiding the need for mastectomy. Considering the presence of a persistent inflammatory response within the mammary tissue and the anticipated abrupt reduction in breast volume, the risk of delayed postoperative hemorrhage was deemed significant. As a result, immediate breast reconstruction, including implant placement, was deferred to minimize surgical complications.

Based on the insights from this case, active surgical intervention should be considered once patients present with any of the following manifestations: persistent pain, alterations in breast contour or firmness, or imaging evidence of capsular nodule enlargement or newly developed calcifications. For patients without these features, a latent phase of hematoma progression may exist, often lacking specific radiologic characteristics. We also recommend surgical intervention with complete capsular excision during this stage, indicating that the optimal timing for surgery may precede the onset of clinical symptoms. Such an approach not only minimizes the risk of further progression of hematoma and related complications, but also allows for immediate breast reconstruction in patients without hematoma or with limited hematoma involvement, thereby improving overall quality of life.

This report presents a single case, and further large-scale studies are necessary to accurately determine the incidence and pathogenesis of hematoma. Moreover, the lack of serum PAAG concentration assessments prevented quantification of the toxic exposure levels.

## Conclusion

The mural organizing hematoma adherent to the capsule, potentially attributable to chronic material-induced erosion and progressive vascular injury, may represent a distinct complication. Given the limitations of radiological differentiation, exploratory surgery is warranted in symptomatic patients for definitive diagnosis. For asymptomatic patients, proactive surgical management may also represent a beneficial option.

## Author contributions

Houhuang Qiu and Qian Liang contributed to the initial drafting of the manuscript and were responsible for the acquisition and processing of clinical images. Xiang Zhou and Fuqiang Pan provided critical revisions and approved the final version of the manuscript.

## Informed consent

Written informed consent for publication was obtained from the patient.

## Funding

This work was supported by grants from National Natural Science Foundation of China (No. 81760344).

## Declaration of competing interest

The authors declare that they have no known competing financial interests or personal relationships that could have appeared to influence the work reported in this paper.
